# Effectiveness of fidaxomicin in preventing recurrence after initial community-associated *Clostridioides difficile* infection

**DOI:** 10.1017/ash.2026.10379

**Published:** 2026-04-20

**Authors:** Adam Hawco, Christopher Myers, Runda Dahhan, Christine Hurley, Edwin van Wijngaarden, Christina Felsen, Ghinwa Dumyati

**Affiliations:** 1 Center for Community Health and Prevention, University of Rochester Medical Center, Rochester, USA; 2 Departments of Environmental Medicine and Public Health Sciences, University of Rochester Medical Center, Rochester, USA; 3 Department of Medicine, Infectious Diseases Division, University of Rochester Medical Center, Rochester, USA

## Abstract

Using population-based surveillance from 2020 to 2024, we examined recurrence through 180 days among adults with initial community-associated *Clostridioides difficile* infection (CDI). Fidaxomicin was associated with a 50% lower risk of recurrence compared with vancomycin. These findings are consistent with current guideline recommendations and are relevant to outpatient management of initial CDI.

## Introduction

*Clostridioides difficile* infection (CDI) causes significant morbidity, with recurrent disease representing a major clinical and economic burden across healthcare settings.^1^ Recurrence occurs in approximately 25% of patients within eight weeks after initial treatment^2^ and is associated with increased mortality, reduced quality of life, and increased cost.^3,4^ In 2021, the Infectious Diseases Society of America (IDSA) and Society for Healthcare Epidemiology of America (SHEA) recommended fidaxomicin over vancomycin as first-line therapy for initial CDI episode based on randomized control trials demonstrating reduced recurrence.^3^ Despite updated guidelines, uptake of fidaxomicin has lagged due to cost and insurance barriers.^3,5,6^

Although the CDI incidence has declined over the past decade, an increasing proportion of cases are now community-associated (CA).^2^ As a result, many patients are treated in outpatient settings, where comparative effectiveness data for initial treatment are limited. Trials of fidaxomicin were conducted largely among hospitalized patients, often included individuals with prior CDI, and predated the shift to outpatient management. Real-world studies have similarly focused on hospitalized and high-risk patients, included patients with prior CDI, and assessed recurrence over short follow-up periods.^7–12^ As a result, findings from prior studies may not be generalizable to outpatient management of initial CA-CDI.

We evaluated recurrence after treatment of initial CA-CDI using the Centers for Disease Control and Prevention (CDC) Emerging Infections Program (EIP) surveillance data from 2020–2024 in Monroe County, New York, and hypothesized that fidaxomicin would be associated with a lower risk of recurrence compared to vancomycin.

## Methods

Adults aged ≥18 years with CDI diagnosed between January 1, 2020, and December 31, 2024, were identified through the CDC EIP surveillance. CDI cases were defined by a positive *C. difficile* nucleic acid amplification test (NAAT) associated with a positive or negative *C. difficile* toxin enzyme immunoassay (EIA) result, and clinician decision to initiate treatment. Only initial cases were included, confirmed by the absence of any prior positive *C. difficile* assay in the surveillance records dating back to 2009.

Cases were classified as CA-CDI if the positive stool specimen was collected in an outpatient setting or within the first three days of hospitalization and the patient had no documented overnight stay in a healthcare facility in the 12 weeks preceding specimen collection. Analysis was restricted to cases treated with a full 10–14-day course of vancomycin or fidaxomicin monotherapy. Patients receiving partial, combination, or multiple treatments were excluded. Mortality data were obtained through the New York State Bureau of Vital Statistics. Cases that died within 30, 56, 90, or 180 days were excluded for the corresponding outcome windows.

Demographic characteristics, comorbidities, prior antibiotic exposure, location of treatment (inpatient vs outpatient) and *C. difficile* toxin EIA results were obtained from standardized case report forms.^1,2^ Prior antibiotic exposure was defined as receipt of any systemic antibiotic in the 12 weeks preceding the diagnosis. We used descriptive statistics to compare characteristics between treatment groups. Recurrence was defined as a positive NAAT or toxin EIA assay and was assessed at 30, 56, 90, and 180 days following completion of treatment. Diarrhea at recurrence was not captured by the surveillance. We assessed differences in recurrence and patient characteristics using bivariate analyses and used Poisson regression models to identify factors independently associated with recurrence, with treatment type (fidaxomicin vs vancomycin) as the primary exposure. Covariates were selected based on clinical relevance and significance in bivariate testing and included sex, age group, race, toxin EIA result, treatment setting, proton pump inhibitor use, H2 blocker use, prior antibiotic use, chronic kidney disease, chronic obstructive pulmonary disease, diabetes, malignancies, and inflammatory bowel disease. Statistical analyses were performed using R version [4.4.2]. This study was deemed exempt by the university institutional review board.

## Results

Among 1,216 initial CA-CDI cases, most were managed in outpatient settings (80.9%). Overall, 1,180 (97%) had documented diarrhea and 150 (7.4%) were treated with fidaxomicin. Case characteristics by treatment group are summarized in Table 1. The median age was 60 years (interquartile range [IQR]: 42–73), 64.6% were female. The majority were White (80.2%), followed by Black (9.0%); 5.8% were Hispanic. Characteristics were generally similar between fidaxomicin, and vancomycin treated cases, although fidaxomicin-treated cases were younger overall and more likely to have inflammatory bowel disease. Characteristics of cases by recurrence at 180 days are summarized in supplemental Table 1. Recurrence varied by age and was more common among cases with positive toxin EIA and chronic kidney disease, but less common among those initially treated in the inpatient setting.

**Table 1.** Characteristics of initial community-associated *Clostridioides difficile* cases by treatment group

Characteristics	Vancomycin N = 1,066 n (%)	Fidaxomicin N = 150 n (%)	*P* Value ^¶^
Sex			.14
Female	689 (64.6)	87 (58.0)	
Age groups (years)			.03
18–39	249 (23.4)	36 (24.0)	
40–59	249 (23.4)	49 (32.7)	
60–84	486 (45.6)	60 (40.0)	
≥ 85	82 (7.7)	5 (3.3)	
Race			.10
White	854 (80.1)	121 (80.7)	
Black	97 (9.1)	13 (8.7)	
Hispanic	61 (5.7)	9 (6.0)	
Other	54 (5.1)	7 (4.6)	
Positive *C. difficile* toxin enzyme immunoassay	321 (30.1)	49 (32.7)	.59
Initial treatment inpatient	200 (18.8)	32 (20.3)	.52
Proton pump inhibitor use (prior 12 weeks)	382 (35.8)	58 (38.7)	.56
H2 blocker use (prior 12 weeks)	156 (14.6)	14 (9.3)	.10
Antibiotic use (prior 12 weeks)	420 (39.4)	60 (40.0)	.96
Diabetes	188 (17.6)	27 (18.0)	1.00
Malignancy	155 (14.5)	20 (13.3)	.79
Chronic obstructive pulmonary disease	243 (22.8)	40 (26.7)	.34
Chronic kidney disease	142 (13.3)	13 (8.7)	.14
Inflammatory bowel disease	62 (5.8)	16 (10.7)	.04

^¶^ *P* values were calculated using χ^2^ tests for categorical variables.

Fidaxomicin was associated with lower recurrence than vancomycin across all follow-up intervals, beginning at 30 days (5.3% vs 13.3%) and persisting through 180 days (9.4% vs 19.2%) (Table 2). Mortality during follow-up was uncommon (≤ 3.9%). In adjusted analyses, fidaxomicin remained associated with lower recurrence risk compared to vancomycin with adjusted risk ratios of 0.44 (95% CI: 0.24–0.74) at 30 days, 0.54 (95% CI: 0.24–0.84) at 56 days, 0.44 (95% CI: 0.24–0.84) at 90 days, and 0.44 (95% CI: 0.24–0.74) at 180 days. Crude and adjusted estimates were similar, suggesting minimal confounding (Table 2).

**Table 2.** Recurrence of community-associated *Clostridioides difficile* cases by follow-up period

Follow-up period	Vancomycin (%)	Fidaxomicin (%)	Crude Risk Ratio (95% CI)	Adjusted Risk Ratio* (95% CI)
30 days	13.3	5.3	0.40 (0.20–0.80)	0.39 (0.20–0.77)
56 days	15.9	8.0	0.50 (0.29–0.88)	0.50 (0.29–0.87)
90 days	18.7	8.7	0.46 (0.27–0.80)	0.47 (0.28–0.80)
180 days	22.0	9.7	0.44 (0.26–0.74)	0.45 (0.27–0.75)

* Adjusted for sex, age group, race, *C. difficile* toxin enzyme immunoassay result, treatment setting, proton pump inhibitor use, H2 blocker use, prior antibiotic use, chronic kidney disease, chronic obstructive pulmonary disease, diabetes, malignancy, inflammatory bowel disease.

CI, confidence interval.

## Discussion

In this population-based cohort of adults with initial CA-CDI, fidaxomicin was associated with a lower risk of recurrence at 30, 56, 90, and 180 days compared with vancomycin. By 180 days, recurrence remained under 10% among fidaxomicin treated cases and approached 20% among those treated with vancomycin. Overall, the recurrence was reduced by half, a substantial difference considering the morbidity and healthcare costs associated with recurrent CDI.^4^

Real-world studies of fidaxomicin have reported mixed results. Many prior evaluations focused on hospitalized populations or included patients with prior CDI episodes, limiting their relevance to initial disease managed in the community.^5,7–12^ In contrast, our study focused on CA-CDI and found a consistent reduction in recurrence with fidaxomicin across all follow up periods.

Several limitations must be noted. Recurrence was defined by repeat positive laboratory testing and may have included colonization rather than symptomatic infection. Information on diarrhea at time of recurrence was not captured by surveillance. NAAT-positive/toxin-negative cases were included because they are frequently treated as CDI in clinical practice; restricting the analysis to toxin-positive cases would have limited statistical power. Conversely, some recurrences may have been treated empirically or tested outside the surveillance network, potentially leading to underestimation. Concomitant antibiotics during the follow-up period were not captured and may have influenced recurrence risk. The relatively small number of fidaxomicin treated cases, likely reflects barriers to access such as cost and insurance coverage and limits power for subgroup analyses.

This study has several strengths. We used population-based surveillance that captured inpatient and outpatient cases across different healthcare systems, enhancing generalizability. By restricting the analysis to initial CDI, we minimized bias related to the higher recurrence risk among those with prior infection. We also accounted for mortality during follow-up, strengthening interpretation of recurrence outcomes.

As CA-CDI represents a growing proportion of cases, reducing recurrence may lessen subsequent clinical and healthcare burden. Our findings are consistent with current IDSA/SHEA recommendations favoring fidaxomicin as first-line therapy for initial CDI and provide real-world evidence particularly relevant to outpatient management of CA-CDI.

## Supporting information

10.1017/ash.2026.10379.sm001 Hawco et al. supplementary material Hawco et al. supplementary material

## Data Availability

The de-identified data and code supporting the findings of this study are available from the corresponding author upon reasonable request.

## Supplementary material

The supplementary material for this article can be found at https://doi.org/10.1017/ash.2026.10379.
