# Supplementary material for: Effectiveness of fidaxomicin in preventing recurrence after initial community-associated Clostridioides difficile infection
Source: Antimicrob Steward Healthc Epidemiol. 2026 Apr 20;6(1):e117. doi: 10.1017/ash.2026.10379 (PMC13104515; doi:10.1017/ash.2026.10379)
Supplement: Hawco et al. supplementary material [file S2732494X26103799sup001.docx]

| **Characteristics** | **No Recurrence**  **N = 930**  n (%) | **Recurrence**  **by 180 days**  **N = 239**  n (%) | **P**  **Value ^¶^** |
| --- | --- | --- | --- |
| Sex |  |  | 0.80 |
| Female | 592 (63.7) | 155 (64.9) |  |
| Age groups (years) |  |  | <0.01 |
| 18 – 39 | 231 (24.8) | 53 (22.2) |  |
| 40 – 59 | 248 (26.7) | 44 (18.4) |  |
| 60 – 84 | 390 (41.9) | 127 (53.1) |  |
| ≥ 85 | 61 (6.6) | 15 (6.3) |  |
| Race |  |  | 0.84 |
| White | 740 (79.6) | 196 (82.0) |  |
| Black | 87 (9.4) | 19 (8.0) |  |
| Hispanic | 55 (5.9) | 13 (5.4) |  |
| Other | 33 (3.5) | 7 (2.9) |  |
| Positive *C. difficile* toxin enzyme immunoassay | 253 (27.2) | 103 (43.2) | <0.01 |
| Initial CDI treatment inpatient | 181 (19.5) | 27 (11.3) | 0.01 |
| Proton pump inhibitor use (prior 12 weeks) | 321 (34.5) | 92 (38.5) | 0.26 |
| H2 blocker use (prior 12 weeks) | 137 (14.7) | 24 (10.0) | 0.08 |
| Antibiotic use (prior 12 weeks) | 368 (39.6) | 95 (39.7) | 0.99 |
| Diabetes | 160 (17.2) | 42 (17.6) | 0.97 |
| Malignancy | 125 (13.4) | 32 (13.4) | 0.99 |
| Chronic obstructive pulmonary disease | 210 (22.6) | 61 (25.5) | 0.38 |
| Chronic kidney disease | 99 (10.6) | 39 (16.3) | 0.02 |
| Inflammatory bowel disease | 56 (6.0) | 20 (8.4) | 0.24 |

Supplementary Table 1. Characteristics of cases with and without 180-day recurrence

**¶ P values were calculated using chi-square tests for categorical variables**
